# Front-Tracking and Gelation in Sessile Droplet Suspensions: What Can They Tell Us about Human Blood?

**DOI:** 10.1021/acs.biomac.4c00753

**Published:** 2024-11-01

**Authors:** Sheila Bhatt, Peter A. Smethurst, Gil Garnier, Alexander F. Routh

**Affiliations:** † Institute for Energy and Environmental Flows, University of Cambridge, Bullard Laboratories, Madingley Road, Cambridge, CB3 0EZ, United Kingdom; ‡ Component Development Laboratory, NHS Blood and Transplant, Cambridge Donor Centre, Cambridge, CB2 0PT, United Kingdom; § BioPRIA, Department of Chemical Engineering, Monash University, Clayton VIC 31688, Australia

## Abstract

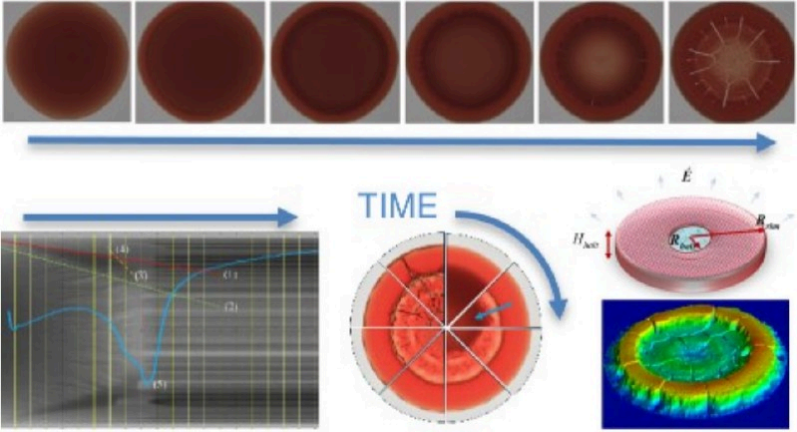

Recently developed imaging techniques have been used to examine the redistribution of human red blood cells and comparator particles dispersed in carrier fluids within evaporating droplets. We demonstrate that progressive gelation initiates along an annular front, isolating a central pool that briefly remains open to particulate advection before gelation completes across the droplet center. Transition to an elastic solid is evidenced by cracking initiating proximal to front locations. The arrested flow of cellular components, termed a “halted front”, has been investigated using a time-lapse analysis “signature”. The presence of a deformable biocellular component is seen to be essential for front-halting. We show a dependence of front-halt radius on cell volume-fraction, potentially offering a low-cost means of measuring hematocrit. A simple model yields an estimate of the gel zero-shear yield-stress. This approach to understanding the drying dynamics of blood droplets may lead to a new generation of point-of-care diagnostics.

## Introduction

Evaporating droplets have received attention from many investigators, looking at flow patterns^1−7^ as well as residue topology^8−12^ and prediction, from pattern interpretation for medical diagnosis to biomechanical properties of constituents.^13−17^ The dynamics of such microdroplets have proved complex, and there is still much to discover. In particular, there has been much interest in applications of biofluid suspensions such as diagnostic devices. Here, we suggest that the dynamics and formed patterns of blood droplet drying are reproducible phenomena that can be used for diagnostic applications.^17−25^

Dried droplets of colloidal fluids have been shown to produce a wide variety of residue patterns.^8,5^ The importance and study of sessile evaporating colloidal droplets originated in industrial fields^26,27^ from paint, adhesives, inks, oil, and 3D printing materials. However, this knowledge base can be leveraged for the study of animal and human bodily fluids and excretions.

While colloids are traditionally defined at a length scale less than 1 μm, particular interest here relates to biofluids often with inclusions at the cellular length scale of a few μm. Multiple investigators have looked for mechanical properties and patterns in various subsets of bodily fluids,^28^ both animal and human: mucous, tears, serum,^29^ etc. The imperative behind this has often been to attempt to isolate and quantify links between disease and the characteristic dried residues of donor samples, for example, saliva,^5^ sweat, urine, and most commonly, blood and blood derivatives.^30^ Previous studies have shown that the deposit topology and dried patterns produced by biofluids such as blood may have some association with specific medical conditions. Many studies have been carried out in an attempt to establish a statistical correlation between the residue-morphologies of blood carrying disease-states or physiological disorders,^15,19,20,31,32^ in order to develop diagnostics for such disorders. However, a consistently applicable statistical relationship has yet to be established, partly due to the inherent variability of so-called “healthy human” samples.

Several studies have investigated the physical origins and evolution of residue morphologies in drying droplets of biocolloids.^8−11^ Evaporation of droplets leads to advection, concentration, and fractionation of constituents, e.g., in red blood cells (RBC), white blood cells, and platelets. Sobac et al.^33^ study the mass loss timeline to show fast evaporation at early times, followed by slow diffusion-controlled evaporation to desiccation. Studies that delineate these different morphological outcomes address the dominance of capillary flow toward the rim or its suppression by Marangoni or other flows offering central packed residues. In blood, apart from the cellular components, dissolved proteins in the aqueous plasma carrier fluid can also be concentrated and accumulated by these flows. Carreón et al.^34^ conducted a texture analysis study of drying patterns to distinguish bovine serum albumin (BSA) in its folded versus unfolded state, showing that in saline (NaCl) carrier fluid it was possible to achieve near-perfect accuracy in identifying solutions containing only 4% of unfolded BSA in a protein solution. In tuberculosis (TB) diagnosis, Pearlman et al.^35^ studied the difficulties of low sensitivity (around 50% despite 100% specificity) for the major method of sputum droplet microscopy in low resource settings. By promotion of Marangoni flow via selected substrate and solvent contact angle, a sample enrichment of 100-fold could be achieved. Picart et al. suggest^36^ that the measurement of the yield-stress of blood may offer a prediagnostic test for systemic sclerosis.

**Figure 1:**
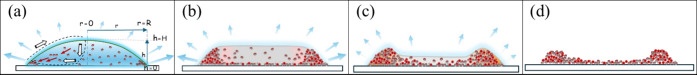
Convex curvature of the sessile pinned droplet results in a higher pressure in the droplet. Concave curvature of interparticle menisci along the pinned contact line results in a local capillary pressure lower than atmospheric, generating a pressure-difference that drives flow. The bulk droplet curvature reduces during evaporation, while the menisci capillary draw persists until the front either halts or reaches the center. (a) Solute accumulating at the contact line, periphery - bulk circulation indicated by white arrows, and red arrows indicate capillary flow to rim, with potential interfacial Marangoni flows. (b) Solute builds a congested front - coffee-ring contrast front. (c) Solute adds more height and breadth to form a “corona”- evaporation reduces carrier fluid level and concentrates proteins which may gel. (d) After evaporation, the solute residue remains wherever the front has halted - morphology of residue is influenced by any front deformation, halt or gelation, or stress relieved by cracking.

Figure 1 illustrates advection and flow circulation within a drying droplet for a coffee-ring morphology that starts to form. Within the droplet, the growth of the packed region can either continue until the center of the droplet is reached or halt allowing vertical evaporation of the remaining central pool.^5,6,33^

**Figure 2:**
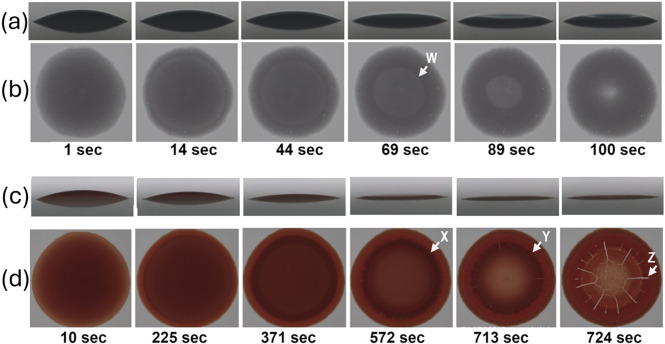
Comparison of front behavior in drying of polystyrene versus blood droplets. Polystyrene: (a) side and (b) top view of rigid 6 μm spheres in H_2_OD_2_O at 40% volume fraction, droplet volume 0.1 μL, contact angle (CA) 17°. Blood: (c) side and (d) top view of deformable 6–8 μm red blood cells (RBC) in plasma at 35% volume fraction (hematocrit or HCT), droplet volume 0.3 μL, CA 14°. Side view has a 2° camera tilt. Annotations (in white) W, X: contrast fronts, Y: halted front, Z: radial crack.

The classic “coffee-ring” deposit has been examined by Deegan,^37^ Fischer,^1^ and Routh^26^ and others in nonbiological colloids. The “coffee-ring” results from a packed congested bed of nonvolatile particulates accumulated from the droplet edge to an inward-growing front of this self-assembled region. The visual appearance of the congested “coffee-ring” regions are captured as distinct contrast-fronts in Figure 2a,b for synthetic polystyrene particles and Figure 2c,d for biological blood macromolecules.

The velocity gradients and halting of self-assembled congested fronts are major features governing the final residue morphology. In the presence of deformable cells or biomechanically responsive constituents, extended evaporation is reported.^38^ These two features suggest that current models which predict the droplet evaporative-residue morphology of rigid spheres may not be easily generalized to blood.^39^ A key question has arisen as to why deposition-fronts for blood have been observed to halt before the droplet center,^40^ where a wet pool of carrier-fluid remains. Previous studies have suggested that gelation may occur from the outer rim of the droplet,^33^ propagating inward. Other authors have considered that a plug of gelled material forms in the central portion of the droplet.^31^

To investigate the question of front-halting, we have developed a front tracking technique to composite entire time-lapse sequences. We have used this to address nonbiological macromolecule droplets as well as simplified blood suspensions, whole blood, and a commercial blood product in which the red blood cells have been modified into spherocytes. Our technique tracks both rim-ward, coffee-ring flows and the inward concentration of particulates. We show how this yields the stages and velocities of the encroaching front and identify its temporal and spatial arrest.

## Experimental Section

The apparatus is fully described elsewhere.^41^ In brief, the apparatus consists of two digital cameras mounted to provide top and side views of an underlit sample stage on which microliter droplets may be deposited by a guided pipet. The side camera images were processed^42^ to provide the contact angle variation with time. Temperature and humidity in the apparatus were recorded every 60 s throughout each experiment.

### Sample Preparation

Suspensions of red blood cells and polystyrene spheres with a range of solute volume fractions (also called hematocrit or HCT, when applied to red blood cells) of between 19% and 61% were prepared using both washed red blood cells (biconcave discoids) of 6 to 8 μm diameter and monodisperse polystyrene spheres 6 μm diameter (Unibead 6–1–0600, density 1060 kg·m^–3^ Chromatech Research), using a benchtop microbalance (On Balance CT-250), and an automatic 0.1–10 μL pipet (SciPette YM209AL0010969). The carrier fluids used were (a) water density-matched to polystyrene via a H_2_O/D_2_O mixture composed of equal parts of H_2_O and D_2_O (Sigma-Aldrich 151882), (b) phosphate buffered saline (PBS, Dulbecco), (c) 0.9% NaCl solution, (d) Celpresol cell-preservative (Immulab, Australia), and (e) human plasma autologous to the RBCs.

Blood samples were prepared from fresh blood obtained from registered donors (anonymised to the study) who had consented for authorized research as part of their standard donation process, in collaboration with NHS Blood and Transplant Component Development Laboratory, Cambridge Biomedical Campus, U.K., and also under informed written consent from donors at BioPRIA, Monash University, Melbourne, Australia. A supply of commercially obtained spheracized red blood cells in a plasma substitute with bovine serum albumin (Securacell, Immulab) was also used. Blood and Securacell samples were sourced for three different blood groups: A, B, and O (all rhesus-positive). Suspensions of human blood cells were prepared from venous blood samples drawn into ethylenediaminetetraacetic acid (EDTA) -treated vacutainers, centrifuged at 1000 RCF for 3 min into three layers: a top layer of straw-colored plasma, a middle layer termed “buffy-coat” containing white blood cells, and a lower layer, which is known as the “pellet”, which contains the packed red cells.

The RBC pellet and plasma layer were extracted into separate tubes. The RBCs were washed in phosphate-buffered saline (PBS), and recentrifuged for a total of three washings. Thereafter the washed RBCs were extracted from the bottom of the centrifuge tube to minimize the plasma component of the stock RBC pellet created. The separated plasma was also recentrifuged at 5000 RCF for 5 min and extracted from the top of the centrifuge tube to produce platelet-poor plasma (PPP). The hematocrit of the stock pellet was measured using both a SYSMEX system and via standard hematocrit tubes centrifuged at 5000 RCF for 3 min before measurement. Aliquots of the required hematocrit (HCT) were made up from the stock pellet and autologous plasma using a 10–200 μL autopipette. All samples and stock were stored in sealed tubes at 4 °C prior to use. Before and during use, samples were left to equilibrate to room temperature for 30 min and gently agitated for 60 s using a benchtop agitator (MS1 minishaker) before deposition.

Experimental data acquisition was performed as previously described^41^ by pipetting a droplet of known volume (1.5 μL) onto a selected substrate and time-lapse tracking of deposition and advection was recorded. A cleaning regime of blowing each substrate with dry nitrogen was employed. Glass substrates from different sources were used to provide a range of initial contact angles (West Lab: 18–30°, clarity: 25–40°, polysine: 20–35°, Academy: 8–16°, RS: 6–14°, Epredia: 20–30°).

### Imaging, Microscopy, and Profilometry

Time-lapse image capture was conducted at 5 s intervals throughout the experiment, with image-capture terminating once no further changes in the image could be detected. Typical sequences of images for blood and polystyrene are shown in Figure 2. The captured images were analyzed in three ways. All three droplet analysis approaches derive from the same raw time sequence capture but provide corroborating perspectives on the droplet evaporative behavior.

Microscopy was conducted using a video-enabled Nikon TS-2 microscope, and laser-profilometry was carried out using an Olympus LEXT OLS-5000 confocal laser profilometer.

### Analysis 1: Front-Tracking Image

**Figure 3:**
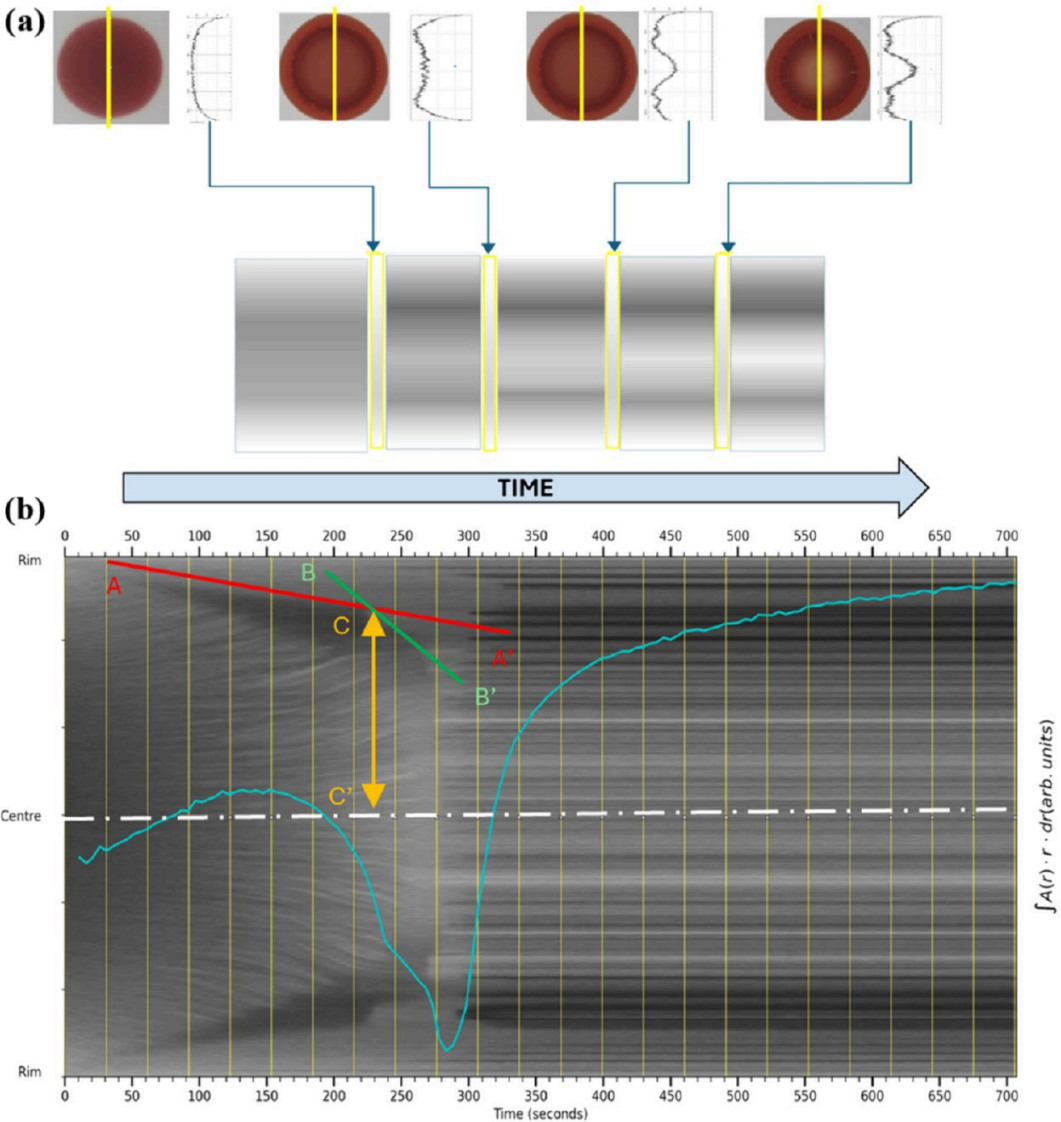
Schematic of front-tracking analysis: (a) top-camera diameter chosen, pixels sampled and added as a 1D column in the front-tracking array, producing a grayscale image (b) that shows the history of contrast-motion within the droplet. Several features are apparent in the trace, representing front formation, motion, possible gelation and cracking of the droplet. The yellow vertical lines on the image identify 30-s intervals, from which a pixels-per-second calibration may be derived. Red and green lines represent front-velocities. Any halting-front position may be determined by a geometric construction that identifies the point in time that the front sharply changes in velocity. Here, a line (A–A′, in red) is fitted to the diagonal contrast-boundary representing the outer front’s initial slow motion. A second line (B–B′, in green) is fitted to the diagonal contrast-boundary representing the rapid depletion of the outer-front at later times. The intersection of these two lines is defined as the “halting front time-point”, and the radius of the halted front can be measured using a vertical projection (C–C′, in yellow) onto the droplet center-line (white dash-dot). The blue line is a plot of the area integral of absorbance over the same time scale, showing the validity of the absorbance method up to the point of front halting.

The first analysis method compiles a representation of the droplet history as the evaporative morphology evolves by using the steps illustrated in Figure 3a. For each sample, a front-tracking image is produced by extracting the raw pixel intensities captured at each time step, across the selected droplet diameter, and compiling these into a single “gray scale” image, as in Figure 3b, that can then be loaded into ImageJ for line-fitting and where measurement tools can be used to calculate the gradients and intersections required, as annotated in Figure 3b. This technique is described below.

### Front-Tracking Image Compilation and Interpretation

In this work we introduce a technique to composite the entire “droplet history” into a single front-tracking image, allowing interpretation of front velocities. This technique allows any time-lapse image-capture sequence to systematically track accumulation and fractionation fronts, providing a means of calculating their velocity. Visually, the optical fronts can be perceived by the human eye as contrast-boundaries but are often indistinct to algorithmic identification when numerically comparing intensities across a sample. In order to identify front-positions, the center and radius of the droplet in each image were identified using ImageJ’s “Analyze” function, and the profile of pixel intensities measured along a line drawn across the diameter of the droplet recorded into an array of pixel-dimensions [1 × droplet-width]. This array was in turn placed into an accumulation array of dimensions [pixel-diameter × experiment-duration], as shown in Figure 3a, and the process repeated for each image in the time-sequence. The accumulation array thus forms a 2D grayscale image showing the variation of intensity across the droplet versus time as in Figure 3b. The front-tracking images generated using this technique allow straightforward comparison of the flow-field and stress-release behavior without having to examine each individual frame, allowing object flow and front velocity to be directly assessed.

The motion of fronts and other features within the droplet are exposed in this visual rendition of the “droplet history” image as diagonal lines whose gradient equals the feature velocity; an example measuring the outer-front velocity is annotated on the example front-tracking image in Figure 3b. It is therefore possible to estimate the velocity of particles and particle-aggregates as streamlines advecting toward the dark congested regions. Gradients A–A′ and B–B′ delineate the outer contrast front of the dark congested region and their intersection represents the time-point of the “halted front”. Once this intersection at C is identified, the radius of the “halted front” may be measured directly by the vertical line C–C′ projected onto the droplet center line. As the droplets are usually not perfectly axisymmetric, the feature treatment can be repeated below the droplet dotted center line, indicating experimental variance.

Figure 3b also shows a solid blue line overlaid on the front-tracking image representing the time variation of the total area integrated absorbance measured during the experiment. In blood, this exhibits a minimum, which we ascribe to the point of complete gelation. This minimum can provide a reference time for events. The horizontal axis can therefore give the duration of the progressive gelation from the front to the center.

### Analysis 2: Droplet Radial Profiles

**Figure 4:**
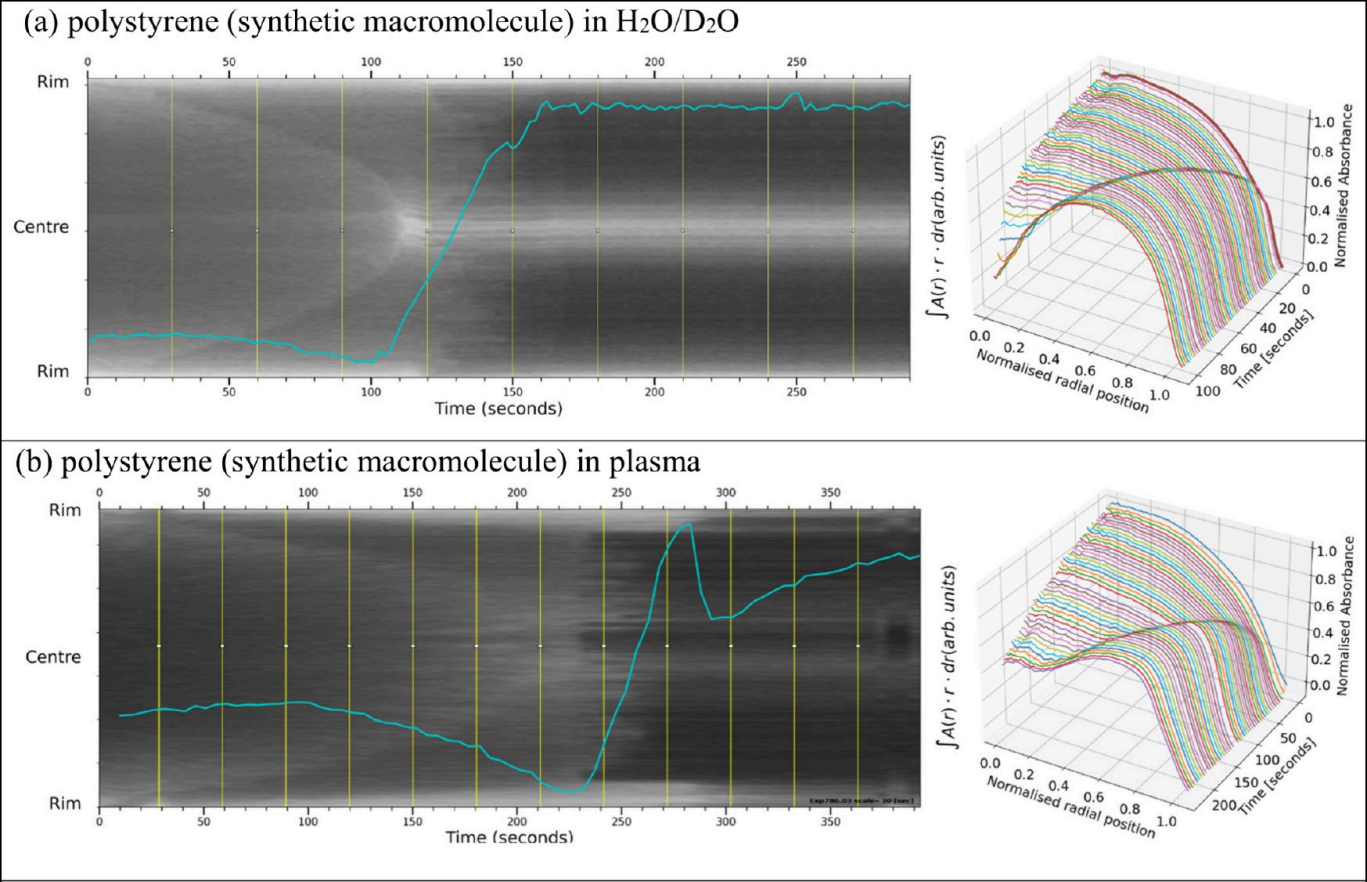
Front-tracking images and profile surfaces of polystyrene particles in water and plasma carrier fluids: (a) 6 μm polystyrene beads (40% HCT, volume 0.1 μL CA 14°) suspended in density-balanced H_2_O/D_2_O; (b) polystyrene spheres (35% HCT, volume 0.2 μL, CA 22°) in human platelet-poor plasma. In both plots, the self-assembly and inward growth of the contrast front are visible, representing the saturated congested particles advected to the droplet edges under the influence of capillary flow. In (a), the contrast-front progresses smoothly toward the center with no outer halt-radius, no “blunt-nose” is visible. In (b), features similar to (a) are seen, but accompanied by more evident striations at a later time.

The second analysis method uses the Beer–Lambert law^43^ in eq 1 to process the advection of the colloids in the droplet,^41^ yielding the eventual residue topology and congested volume evolution normalized to the droplet starting path length.
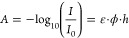
1where *A* is the absorbance, *I* is the transmitted light intensity, *I*_0_ is the incident light intensity, ε is the extinction coefficient, ϕ is the nonvolatile volume fraction of the sample, and *h* is the path length of light passing through the suspension. The absorbance of the carrier fluid was taken as negligible,^41^ as was any change in the material’s intrinsic extinction coefficient (during the valid period where the total absorbance integral was largely constant).^41^ ImageJ’s concentric-circles plugin was used to obtain the averaged radial absorbance values for input to eq 1, generating a 3D time-series surface, as shown in the right-hand side of Figure 4. Errors in the values of the final calculated residue topology were estimated to total ±9%.^41^

### Analysis 3: Total Absorbance Area Integral

**Figure 5:**
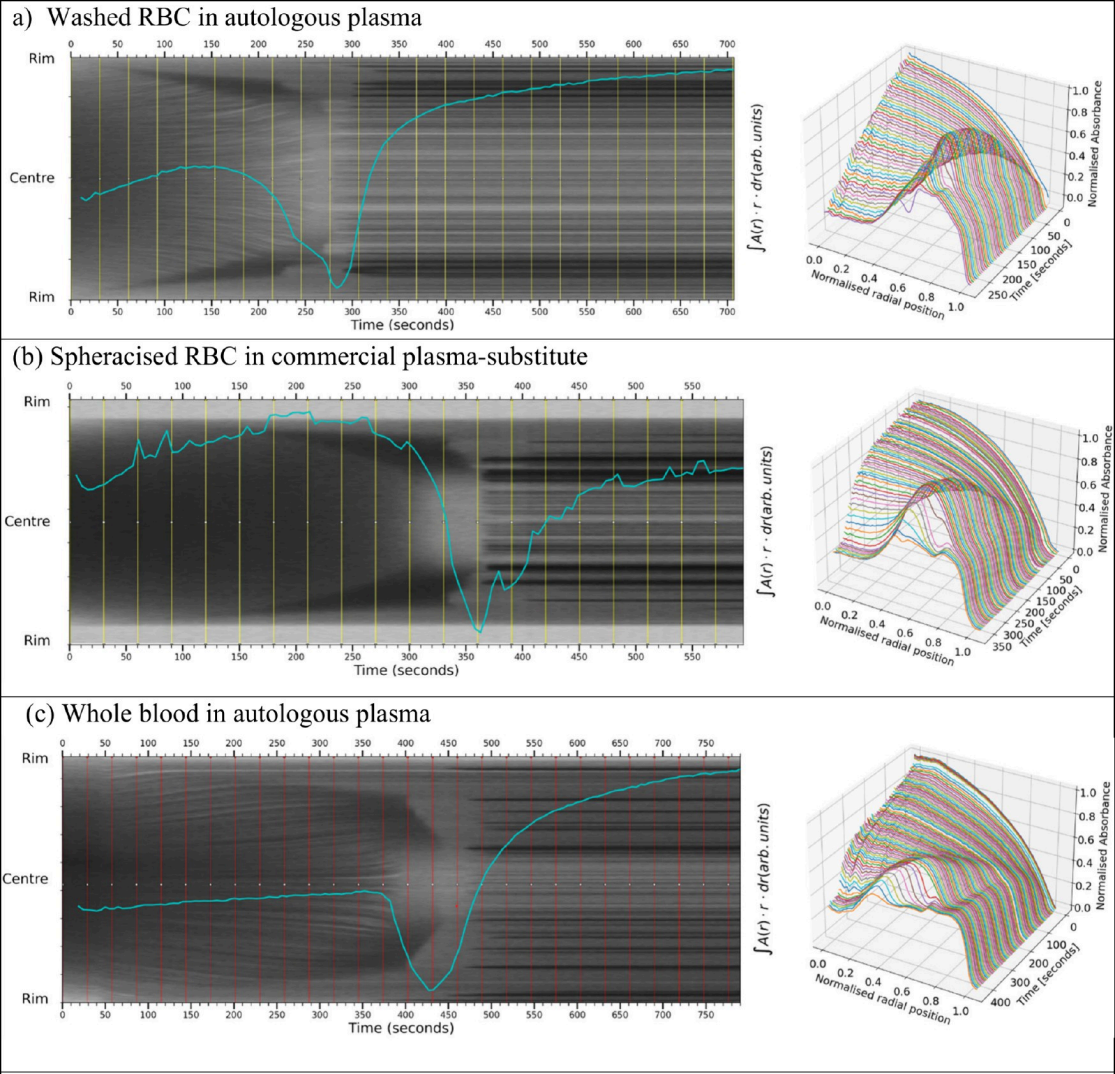
Front-tracking images of blood cells all suspended in plasma or substitute: (a) red blood cells resuspended in autologous platelet-poor plasma (35% HCT, volume 0.25 μL CA 14°), (b) spheracised Securacell cells suspended in commercial plasma-substitute (33% HCT, volume 0.2 μL CA 33°), and (c) whole blood in platelet-rich autologous plasma (47% HCT, volume 0.31 μL CA 24°). In all plots, the establishment and motion of the contrast front are clearly visible, representing the built-up mass of saturated congested particles advected to the droplet edges under the influence of capillary flow. In (a), the outer “halting” front feature is visible at around 240 s. Divergent advective flow lines can be seen in the central region up until 240 s, indicating sufficient carrier fluid remains for the last advection and entrapment of red cells before depletion and complete gelation. The “blunt-nosed” profile at the center occurs at the end of advection. In (b), “halt” features similar to (a) are visible, but no object tracks are visible in the central region, indicating cells move independently rather than clustering. In (c), features similar to those in (a) are visible, although both the final rapid-front-motion period and the “blurred” gelation periods are longer. In all three cases, the “blunt-nosed” signature of a halted-front is clear.

**Figure 6:**
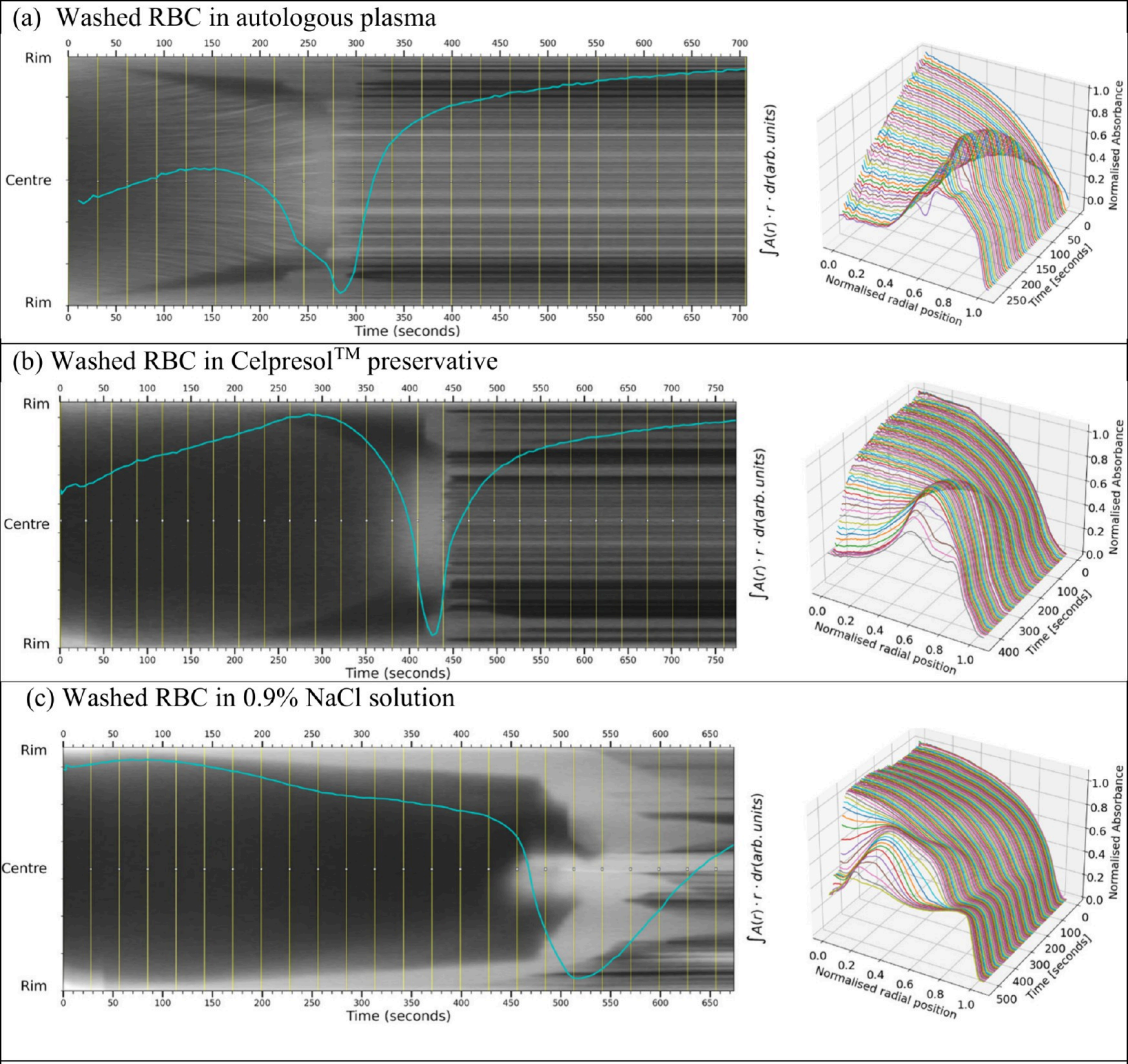
Carrier-fluid variations: Front-tracking images obtained from RBCs resuspended in (a) autologous human plasma (35% HCT, volume 0.25 μL CA 14°), (b) Celpresol preservative (34% HCT, volume 0.28 μL CA 40°), and (c) 0.9% NaCl solution (33% HCT, volume 0.43 μL CA 48°). The front-tracking plots show a halted-front in all three carrier fluids, even in the apparent absence of plasma.

The third analysis method generates a value for the evolution of the total absorbance of the droplet at each time step. The technique is described elsewhere^41^ and shown here as an (time matched) overlay line on the front-tracking images in Figures 3, 4, 5, and 6.

As there was no evaporative loss of the nonvolatile and the absorbance of the carrier fluid was taken as negligible,^41^ a constant area averaged absorbance is expected, giving
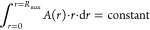
2where *A* is the absorbance, *r* is the radius, and *R*_max_ is the radius of the pinned droplet rim.

### Accuracy and Repeatability

To assess the accuracy and repeatability of the halting-front radius plots, 21 repeated measurements in five separate experimental runs were obtained on the same concentration aliquot. The criterion used to define the halting-front was the same in all measurements and was as described in Figure 3b. Excluding one outlying measurement that contained a dense clot, the results show an average halting-front position, normalized to 0.75 of the droplet radius for this aliquot, with a standard-deviation of 0.03, indicating a coefficient of variation over the 21 repeats of 0.04. Systematic errors from the apparatus were previously calculated,^41^ and these are indicated via error bars in Figures 10 and 11.

## Results

### Polystyrene

The front-tracking image for rigid polystyrene spheres suspended in H_2_O/D_2_O shown in Figure 4a displays a single contrast-front moving progressively toward the center of the droplet diameter, to a bright spot that persists as a horizontal bright pixel intensity line of fixed position. The radial profile plot (shown in 3D) demonstrates that the packed bed progresses to the center of the droplet, showing smooth advection of the particulates. The area integral shows a fairly uniform trajectory with a dip before a rapid rise, possibly due to air ingress into the residue, as previously stated.^41^

No individual aggregated object tracks are visible, indicating that the polystyrene spheres move independently. In Figure 4b, where human plasma was the carrier fluid, the 3D radial profile shows that the packed bed forms a central higher plain, and the advecting particulates have not moved fully out of the center into the coffee-ring. However, the continuous front movement dissipates at a later time, leaving an inconclusive front-tracking image, which suggests that the suspension in plasma may gel, but not sufficiently to cause a definitive halting-front.

### Blood

In comparison with the simple polystyrene case, the front-tracking image in blood yields a more complex picture. The front-tracking images for blood are shown in Figures 5 and 6 accompanied by radial plots and overlaid with a blue line showing droplet absorbance area integral with time.

The front-tracking image obtained from red blood cells in autologous plasma, Figure 5a, shows several distinctive features. In the early part of the image, the establishment of a dark contrast-front is noted, moving slowly toward the droplet center. In this early period, the tracks of objects in the central region moving toward and becoming part of the inside edge of the contrast front can be seen, forming dark “wedge-shapes” in the front-tracking image which broaden with time, corresponding to the growth of a dark annular region in the captured top-images, and showing the inner-front is growing at a faster velocity than the outer-front. These moving objects are approximately ten times the size of a red blood cell and are likely to be blood-cell aggregates known as “rouleaux”. The gradients of these object tracks (and therefore their velocities) are seen to increase with time. Within the dark “wedge-shapes” of congested packed cells no motion-tracks are discernible.

When these wedge-regions stop broadening, there is an abrupt increase in the gradient of their outer edges as the front recedes, terminating in a blunt “square-ended” profile at a fixed distance from the center of the droplet. This termination is the characteristic signature of a “halting” or “halted-front”.

Immediately following this blunt termination, a “blurred” feature is seen coinciding with a minimum in the total area-absorbance integral. This feature typically has a duration of approximately 30 s. Following this blurred feature, regular static horizontal lines appear, indicating the establishment of a fixed crack-pattern suggesting the central region of the droplet now has insufficient unsequestered fluid available to provide advective flow. All of the object velocities after this point are zero.

Figure 5b shows results from Securacell cells (spheracized human red cells) suspended in commercial plasma substitute. The front-tracking image shows a characteristic “halt” feature similar to Figure 5a, and a general pale divergence arising from depletion of the remaining carrier fluid. However, for Figure 5b, the central area of the early time image shows little sign of object-motion tracks, indicating that the spheracised particles move independently rather than clustering, whereas for Figure 5a,c, object tracks are observed, suggesting these cells move as aggregate “rouleaux” rafts.

Results from whole blood are shown in Figure 5c, and they show clear signs of clustering. The front-tracking image once again shows the square-ended feature characteristic of a “halt”, and some similar features to both the washed RBC and Securacell samples.

Unlike the rigid polystyrene suspensions, all of the accompanying radial profiles show that the accumulation fronts in blood exhibit a degree of deformation or collapse postformation. The time-matched total area absorbance profile (from eq 2) is overlaid on each of the “front-tracking” images. In each blood case these show a distinct sharp minimum just prior to increased total absorbance, which suggests air ingress. All the blood cases exhibit unchanging horizontal intensity lines thereafter, confirming a settled crack pattern that can be interpreted to show the relieved stress in the pinned droplet.

### Carrier Fluid

Figure 6 shows tracking images and absorbance profiles for RBC dispersions in three different carrier fluids. Figure 6 suspending red blood cells from the same donor prepared at the same HCT. The carrier fluids are (Figure 6a) autologous plasma, (Figure 6b) Celpresol (commercial blood-preservative), and (Figure 6c) 0.9% NaCl solution. All images show a change in contrast front accompanied by the decline in the absorbance area integral overlay toward a minimum. In Figure 6a, the wedge shapes of the halting front, the blurred region, and the establishment of a fixed crack pattern in the residue are seen. However, there are differences in other fluids; in Figure 6b Celpresol shows a more abrupt front collapse after the “halt” feature, and the front collapse and crack patterns in Figure 6c are distinctly different. In the case of 0.9% NaCl, the signature of a halted front approaches closer to the center of the droplet and overlaps with substantial rim-based cracking. A narrow band of gelled material may well still be present. The corresponding radial profiles once again show a late-time collapse of the front in all cases; the 3D profiles also suggest that late-time behavior in Figure 6b,c is more strongly influenced by deformation of the packed bed of RBCs. The plasma-suspended sample shows a higher central final level. The total area-integral curves, overlaid on each graph, all show the sharp minimum observed in the blood samples measured in Figure 5.

### Microscopy Results

**Figure 7:**
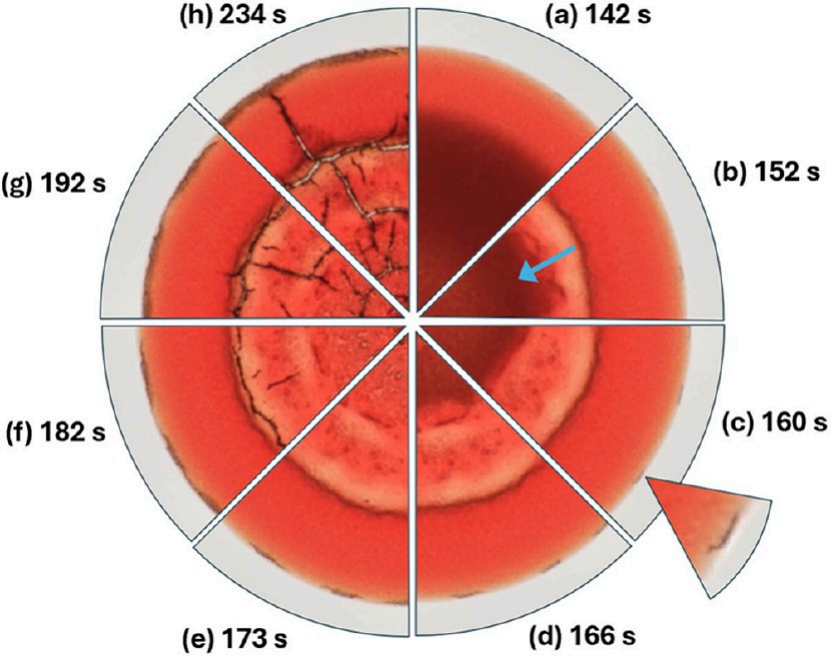
(a–h) A sequence of eight time slices in increasing time postdeposition clockwise from the 12 o’clock position. Starting with (a), the self-assembly of the congested bed, after 142 s. After the front has halted, significant changes take place: the section shows a well-defined contrast-front representing the division between a dark saturated congesting region well-supplied with carrier fluid and a brighter but still wetted near-rim region. (b) 152 s: As the time-period representing the blurred region is entered, a pale front with an ill-defined threshold starts to propagate from the rim-ward side of the contrast-front inward toward the droplet center in the direction of the blue arrow. Simultaneously, a thin dark band forms at the position of the halted front. (c) 160 s: The pale front progresses rapidly toward the center, lightening and “smoothing” out any visible features. (d) 166 s: Front-encroachment continues. (e) 173 s: The pale front reaches the center of the droplet. (f) 182 s: Cracking initiates within the pale material, just inside the thin dark band, and rapidly forms an orthoradial ring. (g) 192 s: Cracks propagate around the inside of the halted front and generally within the material, spreading to the rim region. (h) 23 s: Some time later, radial cracks initiate around the locus of the orthoradial ring-crack and propagate out toward the rim. Inset: ×10 image of filigree edge cracks (figure layout after Sobac and Brutin^33^).

In order to investigate the origin of this “blurred region” of the front-analysis images, droplets of red blood cells in autologous plasma were examined using an optical microscope equipped with video facilities (Nikon Eclipse TS2). Images extracted from this video are shown in Figure 7. The sequence of images encompasses the blurred region in the front-tracking images between the front-halt and final drying. They depict (from rim to center) the same “pie-slice”-shaped section of the droplet at increasing times. As the time-period representing the blurred region is entered, a pale front with an ill-defined threshold starts to propagate from the rim-ward side of the contrast-front inward toward the droplet center, in the direction of the blue arrow. Simultaneously, a thin dark band forms at the position of the halted front. Following this, the pale front progresses rapidly toward the center, lightening and “smoothing” out any visible features. Cracking then occurs, darkening the image.

Increasing the magnification and illumination intensity showed evidence for intact RBCs embedded inside the pale material. No images were captured that indicated cells rupturing. Despite the high resolution and speed of the microscope video, it is unlikely that ongoing cell lysis would be directly captured; therefore, this observation is an interpretation based on the absence of visible cell-membrane detritus. This means the pale material is unlikely to represent a universal lysis front; we do not see obvious evidence that gelation is reliant on cell lysis. The composition of the gelled region is debatable and needs future work. See supplementary video S1 where the sweep of gelation from the halted front to the droplet center is captured.

### Profilometry Results

**Figure 8:**
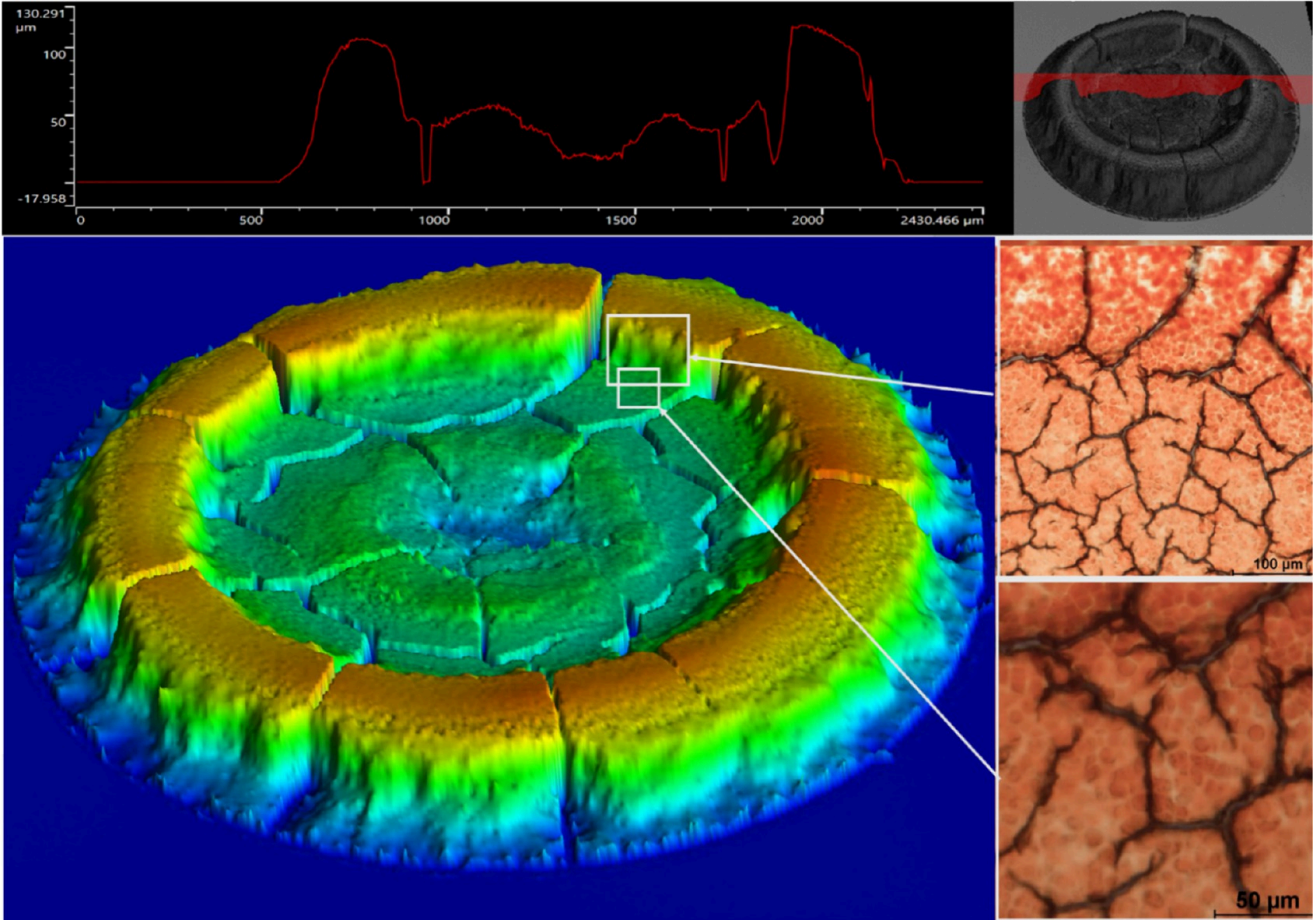
Laser profilometry of droplet residue, washed RBCs in autologous plasma, HCT 35%. We note the sharp vertical inner face of the congested bed on both the main image and a selected profile through the mass. Also, we note some evidence of a smaller inner ring within the central pool (which suggests a “droplet within a droplet”) or possibly fractionation of the denser senescent RBCs. Inset shows photomicrographs of the droplet after end-of-advection, showing intact embedded red cells in the packed-bed and the central plain regions.

These are listed in Figure 8. The radius of the inner face of the coffee-ring residue coincides with the halting-front radius, with orthoradial cracking appearing just inside this front face. It is noteworthy that the inner face of the coffee-ring is very steep, perhaps indicating that the gel front propagating inward from the coffee-ring is a loose saturated gel subsequently dried by vertical evaporation. The height profile highlights the primary arrested coffee-ring deposit and then an inner-ring morphology.

## Discussion

From the results so far presented, we note that the halting-front requires the presence of deformable particles like RBCs, the presence of plasma is insufficient to generate a halt until proteins concentrate sufficiently during evaporation to gel, and gelation is associated with the temporal and spatial locus of a halted front. We now consider three potential influences causing the halting of the front in red blood cell droplets. These are; increased viscous losses, which eventually exceed the capillary draw, the deformable red blood cells plugging the congested bed, and the accumulation of protein concentrations causing gelation. The first cause might simply be attributed to viscous losses rising as the width of the bed increases until the pressure drop from viscous loss exceeds the pressure difference generated from capillary draw. This has been partially addressed by Hertaeg et al.^40^ who consider a worst-case scenario of the bed extending all the way to the droplet center and calculate the ratio of the capillary pressure to the viscous loss given by Darcy’s law, concluding that capillary pressure dominates over viscous loss by 3 orders of magnitude, therefore indicating that the halt cannot be attributed purely to viscous losses. However, this does not address the reducing permeability of the bed due to the nature of deformable cells, which may partially restrict interstices between packed cells as it grows.

Second, we observe here that front-halting in the blood requires the presence of deformable cells, indicating that such plugging might be an important factor in explaining the halt. Another factor related to interstitial plugging is the recession of menisci into the porous bed, when the flow can no longer replenish interstitial fluid at the air surface. This mechanism suggests that capillary draw continues but evaporation is now slowed due to partial saturation of the egress volume of vapor near the recessed menisci, meaning that the bed should continue to grow but at a reducing rate as growth of the bed width accentuates and triggers the further recession of menisci away from the droplet surface. If there is recession of menisci into the porous bed when the flow can no longer replenish interstitial fluid to keep the bed fully saturated to the open air interface, and the droplet is not perfectly axisymmetric such recession would be evidenced by air ingress in selected parts of the droplet rim, which is a possible explanation for the locally restricted rim filigree-cracking we note in Figure 7.

Having established that the halting front cannot be entirely explained by permeability-losses and having considered deformability and protein concentration by evaporation, we move on to consider the evidence for gelation. We observe in Figure 4 that a rigid particulate suspension does not halt and shows a clear smooth front-motion of the packed bed growth right to the center of the droplet. With the same rigid particles suspended in human plasma, Figure 4b also shows a smooth continuous contrast-front movement toward the droplet center at early-time. Only at late-time when plasma proteins may be concentrated by evaporation is there some possible gelation. At late-time there is no obvious abrupt change in velocity of the contrast front in Figure 4b, though the outer edge of the wedge-shaped contrast area does show an initially dark edge receding from the rim replaced by paler encroaching contrast at the periphery. The 3D radial profile suggests that the advecting particles may not have moved as fully out of the center and into the coffee-ring as seen in Figure 4a. We also see some evidence in the form of a diffuse region at late-time that the plasma may be becoming concentrated enough to start to gel. Unlike the blood front-tracking results, there is no observable abrupt change in the front-velocity characteristic of a halt, so gelation from plasma alone (at physiological levels) appears insufficient to cause halting for rigid particulates. However, as the solvent is lost through evaporation and the particulates are packed into the bed, the proteins are also left behind. This implies that increasing viscous losses from decreasing permeability may be occurring in parallel with increasing viscous losses due to the rising viscosity as the solvent is lost.

We also observe in Figure 7 the propagation of a pale region of material sweeping toward the center of the droplet after the front has halted, surrounding and embedding individual RBCs without lysing them, before cracks appear within this pale region. The material of this pale region can be seen to support fractures, so this is evidence that the material may be an elastic solid. The cracking is similar to that observed in many studies of gelled media.^44,45^ Although this study has not identified individual proteins and where these accumulate during droplet evaporation, it seems probable that this material is a gel formed from one or more of the plasma proteins. The major plasma proteins are albumin, globulin and fibrinogen present at about 8% by volume in the plasma.^46^ The presence of EDTA into which the original sample was taken and the plasma platelets having been removed by centrifugation (except for the case in Figure 5c, whole blood) means that it is unlikely that a high proportion of the fibrinogen will have been converted to insoluble fibrin.^47,48^ It should also be noted from the results in Figure 6 that replacing the majority of the plasma with protein-free 0.9% NaCl changes the position of, but does not entirely remove, elements of the contrast-front signature (some plasma will always accompany the red cells from the pellet). Figure 6c shows the abrupt increased velocity of the late-time outer edge of the contrast-front, but also its close approach to the droplet center, and distinctly different static crack intensities after the total area absorbance minimum.

We suggest that gelation is initiated in proximity to three possible loci: the halting-front location (as seen in supplementary video S1 and Figure 7), the rim (seen as localized filigree cracks at early time), and the interstitial gelation tracking the outer front of the congested region. Brutin et al.^33^ offer evidence for gelation starting from the pinned droplet edge; this may also occur here, but the evidence from postgelation cracking is that rim gelation and filigree cracking does not propagate to major fractures until after the front halts, and the area integral has reached a minimum. Bou Zeid and Brutin^49^ indicate a dependency on humidity of the “coronal” (congested region) width and note that higher relative humidity, concomitant with lower initial evaporation rates, lead to the increased size of both the periphery and the coronal region. Humidity in our experiments was controlled to within 10% and continuously monitored. As a result, humidity changes between runs are small, and we would not expect large differences due to this.

Our results suggest that dilution of the plasma and associated plasma-proteins does not suppress the halting front, indicating that while protein-concentration might be involved in deposition and residue-morphology, it alone cannot be the primary reason for the halting-front. A secondary conclusion is that the pattern of cracking phenomena is more dependent on the nature of the carrier fluid, whereas front dynamics depends much more on the particle packing-fraction and deformability. Brutin and Sobac^50^ suggest that substrate-adhesion dominates cracking. Bourrianne et al.^51^ propose that cracking is also dominated by initial volume-fraction.

We therefore conclude that the halting-front is strongly linked to both plugging of packed-bed interstices by deformed RBCs, and by the inner front of the packed bed gelling due to both increased protein concentration and RBC congestion.

Many studies have examined final-residue crack topologies in drying blood,^5,31,50,52^ in which the importance of environmental factors such as humidity are demonstrated. Cracking is accepted as originating from the pinned nature of the contact line that places the droplet in tension under the evaporative loss of solvent,^26^ and is used here as an indicator of solidity. We observe that the front-halting is almost coincident with the locus of cracking initiation in this zone of the gelled material. Even in the platelet poor, protein-diluted carrier fluids, cracks can be seen to propagate toward both the center and the rim of the droplet. In addition, we note at the rim of the droplet that the “filigree” of small black peripheral cracks, which can be radial or orthoradial in orientation, initiate before the front fully halts and propagate in a locally limited and slow manner. These “filigree” patterns appear significantly earlier than the evidence of an elastic solid having developed in the droplet and possibly indicate that the outer rim of the droplet is selectively dewetting because early gelation starts to restrict the uniform flow of solvent necessary to replenish fluid evaporating from the rim. Alternatively, considering the thickness of the peripheral droplet residue, it is possible that these filigree cracks may be due to tiny irregularities in the surface that allow air ingress, forming delamination “fingers” along the rim. We suggest that, for blood, there is a relationship between the dynamics of the halting front and the radial position as well as the time point for crack initiation.

## Correlations with the Halting-Front Radial Ratio

In blood, we observe a correlation of the halting-front position with the initial volume fraction of red blood cells. We consider a model of a congested-front of soft colloidal cells being assembled by radial flows within a droplet architecture where pressure differences are due to curvature.

Many complex fluids, such as network-forming polymers, exhibit fluid behavior only above their yield stress. The near-zero-shear viscosity of whole blood is found to be over twice that of the high shear viscosity.^53^ Whether gelation is triggered at the inside face of the halted front, or progresses either from central gelation out to the front or from rim gelation inward to the halted front (as suggested by Sobac and Brutin^50^), the signature of the halted front can be seen in our technique and is coincident with a transition, shown in Figure 7, of a gelled annulus leading to the arrest of a further volatile flow through to the congested bed.

**Figure 9:**
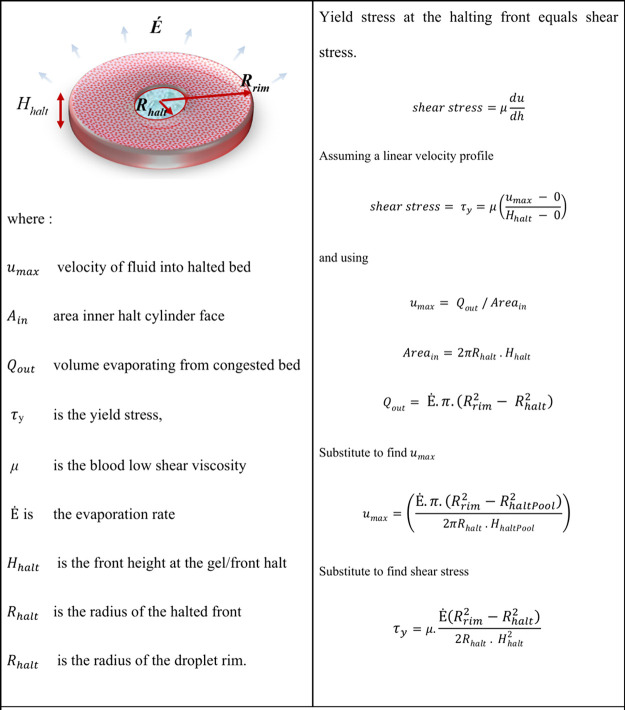
Diagram of the droplet front and halt geometries and a scaling argument for the yield stress of the gel. At the halting front, we equate the shear stress to the yield stress of the gel, giving eq 1. However, the front position still delivers a demarcation between a consolidated outer and via a gelled transition of the inner region. The relative sizes of these regions are indicative of the material yield stress.

Our technique can be applied to the coffee-ring or other morphologies and exposes the key features of the morphology of any sample in order to understand what this implies about the properties of the blood or biofluid constituents and their interaction. One such possibility is around the size of the drying front region, as sketched in Figure 9. Irrespective of where the gelation starts at the center, the rim, or close to the halted front, we can state a simple balance of the quantity of volatiles evaporating from the congested bed equaling the volumetric flow through the front. If we calculate this volumetric demand, we have a measure of the Darcy superficial velocity gradient as we track the height of the front. At the point that the front halts, our experimental measure of the height of the internal face of the front allows calculation of the velocity gradient, which multiplied by viscosity gives the yield stress of the gel.
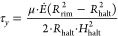
3When this equivalence is satisfied, the central area has gelled and no longer provides liquid to replenish and advecting material to join the self-assembled congested front. This provides a simple experiment to measure the critical yield stress of a blood sample via the ratio *R*_halt_/*R*_rim_. This also indicates that the droplet fronts are dependent on the evaporation rate *Ė*, as found by Bou Zeid and Brutin.^49^

Having shown that the geometry of the halting front is critical to the final morphology and is related to the sample’s intrinsic properties, we investigated what this key “radius-ratio” might tell us about the sample properties. To test this, we performed a series of experiments gathering front-tracking data on human blood samples covering a range of hematocrits and plasma concentrations to investigate possible correlations. By using the front-tracking images, we were able to identify the time points at which to perform the halted geometric analysis, directly measuring the radius of the halted-front normalized to the droplet radius using the analysis described in Figure 3.

**Figure 10:**
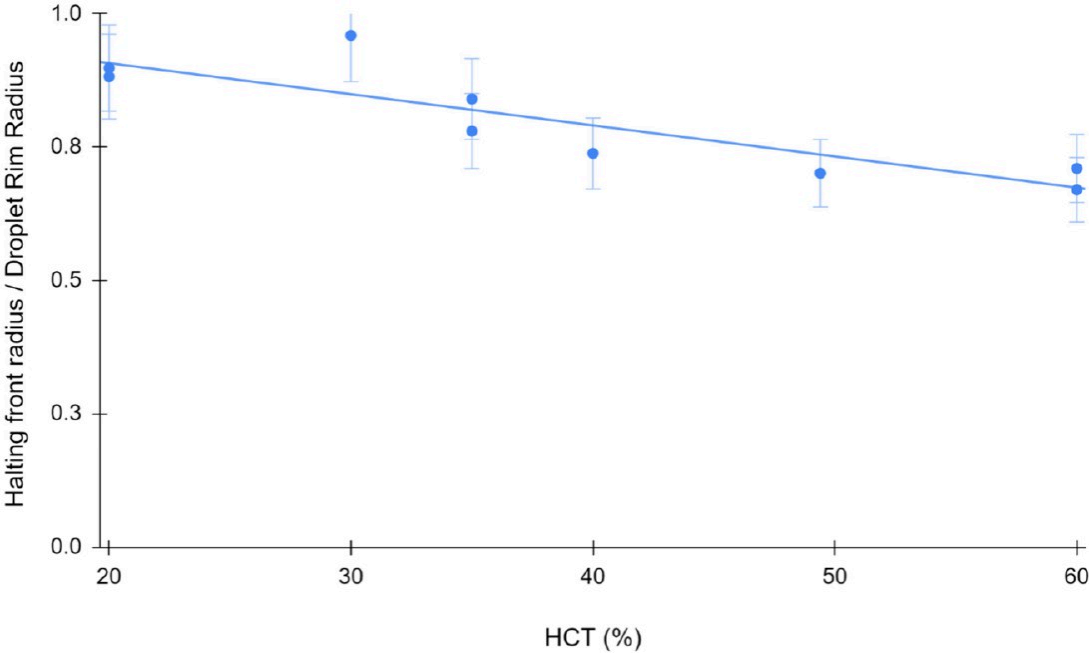
Halting-front radii normalized to a droplet diameter plotted over a range of HCTs obtained from human blood samples, showing a clear negative linear dependence on the ratio with HCT. This indicates that the halted front forms closer to the center as HCT increases.

In Figure 10, the radius-ratio as a function of initial volume fraction exhibits a linear dependence with a negative slope. This means that the halted-front forms closer to the droplet center as the hematocrit is increased. One mechanism that would explain this dependence is that for higher HCT, the total mass of congested material carried to the front for every unit of volatile lost is higher and therefore the congested bed is able to propagate further inward before sealing. When the congested bed reaches a critical shear stress on the cell membranes, these deform, clog, and largely seal the voids in the congested region, preventing further substantial flow and stopping the accretion of cellular material to add to the growth of the congested region.

**Figure 11:**
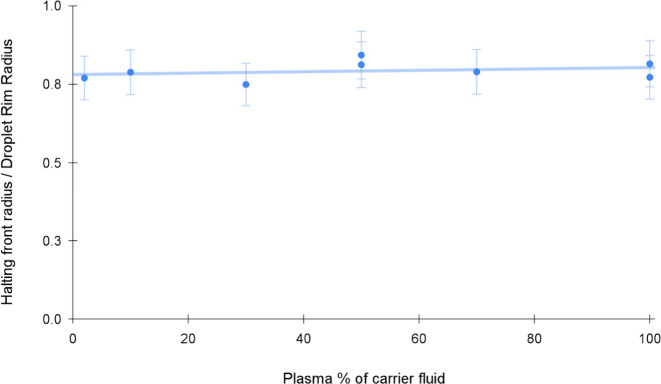
Halting-front radii normalized to droplet diameter plotted against plasma-dilution, human blood, HCT = 35%. The concentration of plasma in the carrier fluid appears to have no substantial influence on the position at which the congested front halts. Theories suggest that the plasma concentration may have an influence on the yield stress.

In Figure 11, the radius ratio as a function of plasma-dilution at fixed HCT indicates negligible dependence of the ratio on plasma-dilution

Figure 11 suggests that plasma concentration (and, by implication, protein concentration) has little effect on the radius at which the front halts. This suggests that the fluid-dynamics are not influenced by the presence of plasma proteins, which is a somewhat surprising result. We hypothesize that the influence on the fluid dynamics is not apparent at early times when the concentration of proteins in the aqueous plasma is <10%. At later times, after evaporation has concentrated these proteins, we see gelation, but by the time this significantly affects the macroscopic rheology, the front is already halting due to the combination of packing and deformation of RBCs as well as gelation.

In contrast, HCT does seem to influence cellular transport. Although it is true that pure plasma with no RBCs forms a ring,^40^ the results in this study indicate that the mechanisms driving front-halting are influenced by the presence and concentration of RBCs. The suggestion of a deformation-and-plugging model of front-halting and sealing is supported by the negligible protein concentration dependence. The plasma proteins alone cannot seal the packed face of the congested front, and diluting them does not suppress front-halting. Varying the bulk initial plasma concentration does not seem to assist or retard the halting. Therefore, we believe RBCs are the essential requirement for halting. However, both Brutin and Sobac^50^ and Mukhopadhyay et al.^54^ suggest that cell-to-cell and cell-to-substrate interaction energies dominate both front-formation and cracking morphologies.

The potential applications of the results of this work are already of significant interest in a number of prediagnostic^55^ and remote-testing areas. One example is a low-cost home application that can show hematocrit.

## Conclusions and Future Work

We present novel experimental results that demonstrate and confirm the existence of a “halt” in front-progression in the drying of human blood droplets that is not present in simple polystyrene suspensions. We show that the halting-front is critically dependent on the presence of biological cells and show that a halting front cannot be generated using inert spheres in human plasma. We further show that the radius at which the front halts normalized by the droplet radius varies in a uniform, predictable manner with the hematocrit, but does not vary with the dilution of plasma in the carrier-fluid of the sample. We show that an annular gelation front forms at the inside edge of the halted front, this propagates inward, turning into an elastic solid in which cracks initiate and propagate, and that the inner rim of the halted front is the location in which orthoradial cracks often initiate. Laser-profilometry of the residue indicates that this inner-front region forms a sharp vertical “cliff” into the central plain in which the final advection is largely driven by vertical evaporation. We infer that fibrinogen may be an important component of this gelled region, and work is currently in progress using IR spectroscopy to examine the chemical makeup of the residue across the droplet radius to investigate this. Our current results suggest that red cells with a degree of flexibility participate in the gelation that creates a halting front. Our observation is that the presence of red cells appears to be necessary for a halting-front to occur and that it is likely that the extreme deformability of RBCs under shear stress participates in reducing the dynamics of flow at the front of the congested bed to below the yield stress of the gelled region. This prompts gelation, which enmeshes red cells in the protein gel matrix. Gelation in this region further changes the flow conditions, triggering a cascade of gelation that propagates from the front region. Finally, the results indicate that the dynamics of blood droplet drying and pattern formation are reproducible phenomena that can be used for diagnostic applications. The observed negative linear dependence of the ratio of halting-front to droplet radius as a function of initial hematocrit suggests that this straightforward geometric relationship could form the basis of a low-cost means of measuring hematocrit.

## Supporting Information Available

Video S1 of gelation-front evolution during drying. Washed human red blood cells resuspended in autologous platelet-poor plasma, HCT 35%. Video shows propagation and halt of contrast-front, motion of clustered RBCs driven by capillary flow, propagation of annular gelation-ring and subsequent cracking initiated around inside of halted-front (MP4)
Video S2 of gelation-front evolution during drying. Washed Securacell blood cells resuspended in isotonic plasma substitute , HCT 35%. Video shows propagation and halt of contrast-front, motion of individual sphericised RBCs driven by capillary flow, annular gelation-ring and subsequent cracking initiated in central region and around inside of halted-front (MP4)
Video S3 of gelation-front evolution during drying. Whole human blood with EDT, HCT 47%. Video shows propagation and halt of contrast-front, motion of clustered RBCs driven by capillary flow, propagation of annular gelation-ring and subsequent cracking initiated around inside of halted-front (MP4)
